# Longitudinal assessment of bone mineral density in prostate cancer patients: comparing metastatic and non-metastatic regions

**DOI:** 10.1007/s10147-025-02711-7

**Published:** 2025-02-07

**Authors:** Takuto Hara, Hanako Nishimoto, Tomoaki Terakawa, Yasuyoshi Okamura, Yukari Bando, Hideto Ueki, Kotaro Suzuki, Yoji Hyodo, Jun Teishima, Koji Chiba, Ryosuke Kuroda, Hideaki Miyake

**Affiliations:** 1 Department of Urology, Kobe University Graduate School of Medicine, 7-5-1, Kusunoki-Cho, Kobe, 650-0017 Japan; 2 Department of Orthopaedic Surgery, Kobe University Graduate School of Medicine, Kobe, Japan

**Keywords:** Prostate cancer, Bone mineral density, Androgen-receptor signaling inhibitors, Androgen deprivation therapy

## Abstract

**Objectives:**

Prostate cancer patients receiving androgen deprivation therapy (ADT) have increased risks of decreased bone mineral density (BMD). However, there are no established guidelines for assessing BMD in patients with bone metastases. The aim of this study was to assess the effects of ADT on bone health by comparing longitudinal changes in BMD between prostate cancer patients with and without bone metastases.

**Methods:**

A single-center observational study was conducted from February 2020 to January 2023 at Kobe University Hospital. BMD at the lumbar vertebrae, total hip, and femoral neck was measured at baseline, 6, and 12 months using dual-energy X-ray absorptiometry. Bones were classified into Metastatic Site (with metastases), Non-metastatic Sites (from patients with bone metastases), and Control (patients without metastases) groups. All patients received luteinizing hormone-releasing hormone antagonists or agonists plus oral ARSI or bicalutamide for 1 year.

**Results:**

Among the 78 patients, 35, 110, and 245 bones were classified into the Metastatic Site group, Non-metastatic Sites group, and Control group, respectively. The Metastatic Site group exhibited significantly higher T-scores compared with the other groups (*P* < 0.001). Repeated measures analysis revealed a statistically significant reduction in T-scores over time across all groups (*P* < 0.001). However, no significant interaction was observed between group classification and time (*P* = 0.817).

**Conclusion:**

The present study demonstrates that BMD changes at non-metastatic sites in patients with bone metastases are similar to those in patients without metastases. Monitoring BMD at non-metastatic sites may provide valuable insights into ADT's effects on bone health in prostate cancer patients.

**Supplementary Information:**

The online version contains supplementary material available at 10.1007/s10147-025-02711-7.

## Introduction

Prostate cancer frequently metastasizes to the bone, with around 10% of patients having bone metastases at the time of diagnosis [1]. Prostate cancer cells express androgen receptors, utilizing testosterone and other male hormones as growth factors [2]. Androgen deprivation therapy (ADT) is the cornerstone of treatment for metastatic prostate cancer. Despite the prolonged survival benefits conferred by ADT [3], the treatment has potential long-term adverse effects, particularly on bone health, necessitating vigilant monitoring [4–6].

The reduction in bone mineral density (BMD) associated with ADT can significantly increase the risk of developing fragility fractures and contribute to mortality [7]. Guidelines in many countries recommend routine bone mineral densitometry to monitor the onset and progression of osteoporosis in patients undergoing hormone therapy [8,9]. While declining trends in BMD over time have been documented in prostate cancer patients without bone metastases [10–12], the assessment of BMD changes in patients with bone metastases—especially those receiving more potent antiandrogenic therapies, such as androgen-receptor signaling inhibitors (ARSI)—remains less standardized [13]. In this single-center observational study, we prospectively evaluated BMD in a cohort of hormone-treated prostate cancer patients, comparing trends between those with and without bone metastases.

## Methods

We enrolled consecutive prostate cancer patients treated at Kobe University Hospital between February 2020 and January 2023. Initial diagnosis included histological analysis, bone scintigraphy, and thoracoabdominal computed tomography to confirm the presence of bone metastases. Throughout their treatment, patients underwent regular prostate-specific antigen tests (every 1–3 months) and received periodic CT scans of the chest and abdomen, the frequency of which was determined by the progression of their disease. Patients who developed castration-resistant prostate cancer within 1 year of starting hormone therapy were excluded. All included patients continued to receive luteinizing hormone-releasing hormone antagonists or agonists in addition to oral ARSI or bicalutamide for a period of one year. The study was conducted in accordance with the principles outlined in the Declaration of Helsinki. Verbal informed consent was obtained from all participants after providing a thorough explanation of the study's objectives, procedures, and potential risks. This approach was approved by the institutional ethics committee at Kobe University (IRB No. B190287).

BMD measurements were conducted at the femoral neck, total hip, and lumbar spine (L2–L4) using a Hologic Horizon A device (Hologic, Inc., Marlborough, MA, USA) prior to hormone treatment, and then at 6 and 12 months thereafter. In this study, the femoral neck on the side without metastases or with minimal metastatic involvement was selected for evaluation based on imaging findings. If both sides were free of metastases, the left femoral neck was consistently used for analysis. Osteoporosis was diagnosed and treated following the 2012 revision of the diagnostic criteria for primary osteoporosis [14]. Osteoporosis was diagnosed and treated following the 2012 revision of the diagnostic criteria for primary osteoporosis, which defines osteoporosis as a T-score of ≤ − 2.5 at the lumbar spine, total hip, or femoral neck. Patients diagnosed with osteoporosis either prior to or within 1 year of starting ADT were excluded from the study. Additionally, patients who were prescribed bone-modifying agents (e.g., bisphosphonates or denosumab) or osteoporosis drugs before or during the study period were also excluded to minimize potential confounding effects on bone mineral density (BMD) measurements. An orthopedic specialist (H.N.) assessed each patient for fragility fractures before and during treatment, utilizing medical history reviews and two-view X-rays of the lumbar region. Patients diagnosed with osteoporosis either prior to or within 1 year of starting ADT were excluded from the study.

BMD values were converted to T-scores and analyzed using repeated measures ANOVA and *t* tests. For this analysis, individual bones were categorized into three groups based on the presence or absence of metastases. The Metastatic Site group included individual bones with confirmed metastases, as determined by imaging modalities such as bone scintigraphy and computed tomography. The Non-metastatic Sites group consisted of individual bones without metastases from patients who had at least one bone with metastasis. The Control group comprised individual bones from patients without any bone metastases.

Post hoc tests were conducted using Bonferroni's multiple test. Statistical analyses were performed using EZR version 1.55 (Saitama Medical Center, Jichi Medical University, Saitama, Japan) [15], with significance set at *P* < 0.05.

## Results

The Supplemental Figure outlines the progression of patient selection. Out of the initial 110 patients, 23 were found to have osteoporosis at the start. Two were subsequently removed from the study due to a diagnosis of osteoporosis within the first 6 months of treatment. We also excluded two patients who transferred out, and five who developed castration-resistant prostate cancer. Finally, 29 patients with bone metastases and 49 patients without were included in the analysis. All metastatic lesions included in this study exhibited either osteoblastic or mixed changes, as identified through bone scintigraphy and computed tomography. Purely osteolytic bone metastases were not observed in the study cohort and therefore not included in the analysis.

Patient characteristics were compared between those with and without bone metastases (Table 1). Notably, individuals with bone metastases exhibited significantly higher PSA levels, Gleason scores, and bone-specific alkaline phosphatase levels, as well as markedly reduced testosterone levels. No significant differences in age, BMI, and the prevalence of diabetes were found between the groups. Of the patients with bone metastases, 19 (64.3%) were treated with ARSI. In the Metastatic Site group, among 29 patients with at least one bone metastasis, a total of 35 metastases were identified in the second, third, and fourth lumbar vertebra (L2–4); total hip; and femoral neck. In the Non-metastatic Sites group, no metastases were observed in 110 sites. These were compared with 245 sites in 49 patients without bone metastases in the Control group.

**Table 1:** Need caption

Variable		PCa without bone mets *N* = 49	PCa with bone mets *N* = 29	*P* value
Age, years (range)		74 (53–87)	71 (52–83)	0.072
BMI (range)		23.4 (17.8–27.5)	22.8 (17.5–29.4)	0.420
Performance status (%)	0	41 (83.7)	13 (44.8)	0.001*
	1	6 (12.2)	14 (48.3)	
	2	2 (4.1)	2 (69)	
Median PSA, ng/mL (range)		10.2 (0.3–302)	269 (0.17–5376)	< 0.001*
Gleason grade group (%)	1 or 2	8 (16.3)	0	0.001*
	3	13 (26.5)	1 (3.4)	
	4	15 (30.6)	17 (58.6)	
	5	13 (26.5)	13 (37.9)	
DM (%)		2 (4.1)	3 (10.3)	0.355
Habitual drinking (%)		11 (22.4)	11 (37.9)	0.194
Current smoking (%)		4 (8.2)	5 (17.2)	0.280
Median BAP, μg/L (range)		12.2 (4.3–26.9)	29.6 (7.2–567)	< 0.001*
Median 25(OH)D, pg/mL (range)		20.4 (4.6–41.2)	17.1 (4.4–47.8)	0.198
Free testosterone, pg/mL (range)		5.1 (2.0–13.4)	3.62 (1.2–8.5)	< 0.001*
Androgen-receptor inhibitor	Bicalutamide	49 (100)	10 (34.5)	< 0.001*
	Abiraterone	0	3 (9.1)	
	Apalutamide	0	16 (55.2)	
CHAARTED volume criteria	No metastasis	47 (95.9)	0	< 0.001*
	Low	1 (2.0)	9 (31.0)	
	High	1 (2.0)	20 (69.0)	
Bone metastatic site	L2	0	9 (31.0)	-
	L3	0	8 (27.6)	-
	L4	0	9 (31.0)	-
	Total hip	0	6 (20.7)	-
	Femoral neck	0	3 (10.3)	-
	Others	0	33 (100)	-

*PCa* prostate cancer, *BMI* body mass index, *PSA* prostate-specific antigen, *BAP* bone-type alkaline phosphatase

^*^Significant

**Fig. 1:**
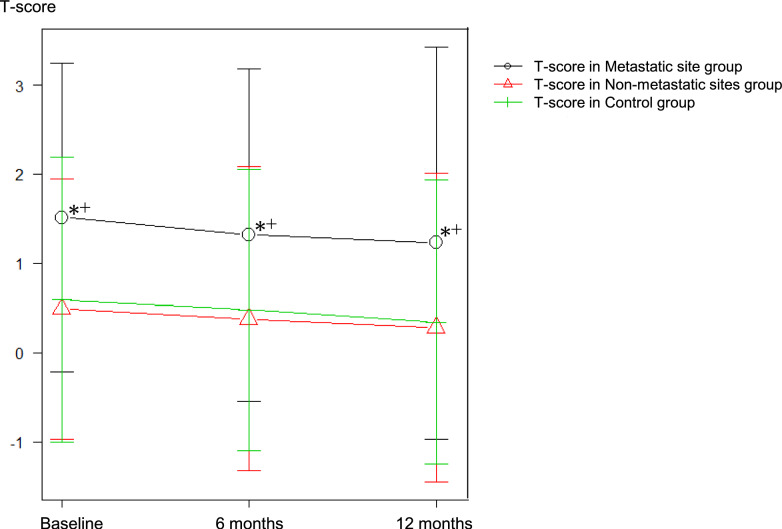
T-score changes over time for the three groups: metastatic site group (black line), non-metastatic sites group (red line), and control group (green line). The metastatic site group consistently exhibited significantly higher T-scores compared to the other groups (*P* < 0.001). Repeated measures analysis revealed a statistically significant reduction in T-scores over time across all groups (*P* < 0.001), though no significant interaction between group classification and time was observed (*P* = 0.817). " + " and "*" indicate significant differences from the Non-metastatic Sites and Control groups, respectively (*P* < 0.05)

Figure 1 shows the bone mineral density at baseline, and 6 and 12 months for each group. The Metastatic Site group had a significantly higher T-score than the other two groups (*P* < 0.001). Repeated measures ANOVA indicated a significant main effect of time (Greenhouse–Geisser corrected *P* < 0.001), suggesting a reduction in T-scores over time across all groups. No significant interaction between group classification and time was observed (*P* = 0.817), indicating that the patterns of T-score change over time were consistent across groups.

**Fig. 2:**
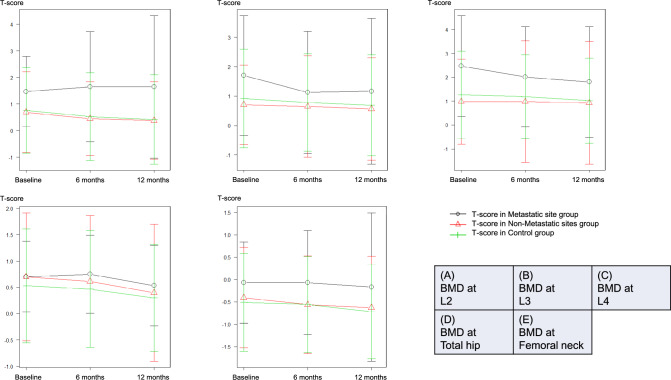
T-score changes over time at L2–4, total hip, and femoral neck across the metastatic site (red line), non-metastatic sites (red line), and control (green line) groups

**Fig. 3:**
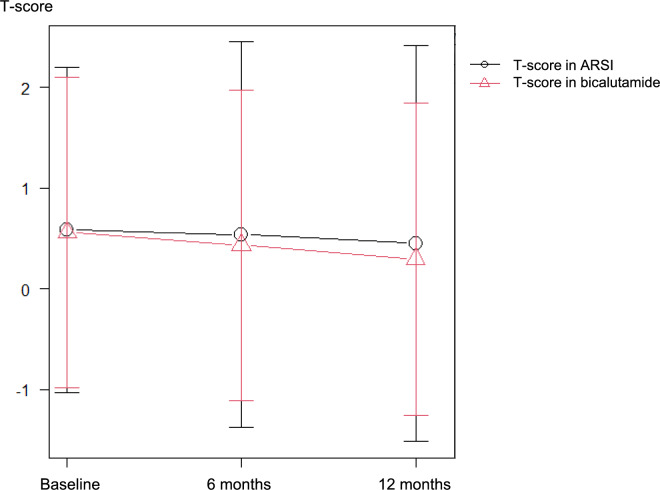
T-score changes over time at non-metastatic bone treated with androgen-receptor signaling inhibitors (ARSI) (black line) and bicalutamide (red line)

Figure 2a–e shows the temporal T-score changes in the L2–4, hip, and femoral neck regions, respectively. The Metastatic Site group had higher T-scores than the Control and Non-metastatic Sites groups; however, there was no significant difference. Figure 3 shows T-score changes over time in relation to ARSI treatment at sites devoid of bone metastases, merging data from the Non-metastatic Sites and Control groups. No significant treatment-based variations were found.

**Fig. 4:**
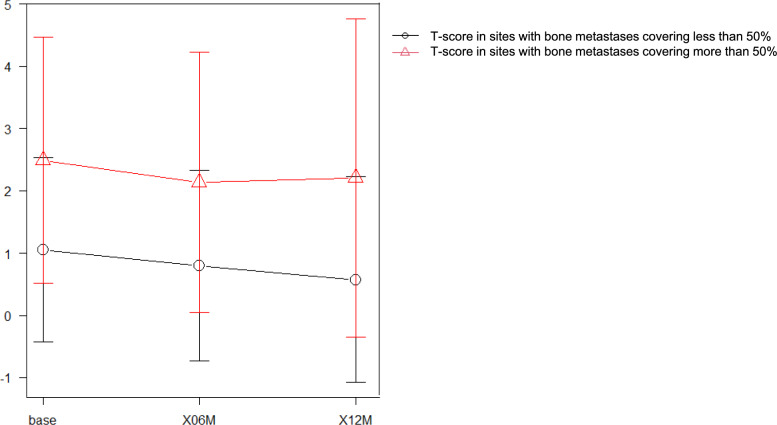
T-score changes over time at metastatic sites. Sites with bone metastases covering less than 50% are indicated by the black line and T-score in sites with bone metastases covering more than 50% by the red line

Of the 35 bone metastases, 11 covered more than 50% of the bone, and 24 covered less than 50%. Sites with bone metastases covering more than 50% of the bone tended to have higher bone density than sites with bone metastases covering less than 50%, as shown in Fig. 4.

## Discussion

Standardized methods for assessing BMD in prostate cancer patients with bone metastases are lacking. Moreover, few studies comparing the rates of bone mineral loss between prostate cancer patients with and without bone metastases have been reported. The present study represents the first attempt to compare longitudinal changes in BMD between non-metastatic and metastatic regions in prostate cancer patients.

BMD assessment typically employs dual-energy X-ray absorptiometry (DXA), focusing on the lumbar spine, total hip, and femoral neck to detect osteoporosis [14]. Prostate cancer frequently metastasizes to the lumbar spine and pelvic bones [16], making accurate bone density measurement difficult. The DXA method utilizes T-scores to evaluate bone density, allowing for comparison against standard values for different measurement sites.

Figure 1 highlights differences in T-scores based on bone condition. Bone metastatic sites have predominantly higher bone density than those without bone metastases, making it difficult to make a diagnosis of osteoporosis based on T-score in patients with bone metastases. On the other hand, T-scores at non-metastatic sites in prostate cancer patients with bone metastases closely mirrored those of sites without such metastases, with comparable trends over time. Our findings strongly suggest that ADT uniformly reduces BMD, regardless of the presence of bone metastases. Repeated measures ANOVA further confirmed a statistically significant reduction in T-scores over time across all groups (*P* < 0.001), while no significant interaction between group classification and time was observed (*P* = 0.817). These results indicate that the patterns of T-score decline are consistent across metastatic and non-metastatic groups, reinforcing the hypothesis that systemic therapy uniformly impacts bone health.

A notable advantage of our comparative methodology lies in its potential to gauge the impact of ARSI treatment on bone density. ARSI is a more potent class of androgen-receptor inhibitors than older antiandrogens such as bicalutamide [13]. Although there are concerns regarding adverse effects on BMD [17,18], the precise impact of ARSI remains to be elucidated [19]. Because ARSI has generally been used as a treatment for prostate cancer with bone metastases, it is difficult to capture the changes in BMD associated with its treatment. A previous clinical trial that examined bone density trends with ARSI reported that bone densitometry had been performed without assessing the extent of bone metastases [20]. Our cohort comprised a limited number of metastatic prostate cancer patients undergoing treatment with ARSI. The rate of BMD decline in these individuals did not demonstrate a discernible deviation from that observed in cases treated with combined androgen blockade, indicating near-equivalence between the two treatment modalities.

As previously reported [21], BMD is higher at sites with bone metastases in prostate cancer, and our study showed a trend towards higher bone density at these sites. Conversely, bone density in regions with < 50% extent of bone metastases showed similar results to those of normal bone. Although bone metastasis determination in our cohort relied on conventional bone scintigraphy and computed tomography rather than PSMA-PET, micrometastases, which are undetectable by conventional imaging modalities, may exert a negligible influence on measured bone density. Future research is needed to assess PSMA-PET imaging as a technique for more accurately identifying bone metastases and their impact on BMD. This could potentially reveal finer details about the interaction between cancer progression and bone health.

Our study has several limitations. First, the relatively small sample size precluded conclusive validation, rendering our findings descriptive. To enhance the validity and generalizability of our findings, future studies should aim for larger, more diverse cohorts and longer follow-up periods to fully capture the long-term effects of ADT on bone health. Second, the study's relatively short duration precluded elucidation of long-term trends. Third, the absence of fragility fracture events in our cohort precludes definitive conclusions regarding the clinical ramifications of reduced bone density in patients with bone metastases. Additionally, the lack of data on bone metabolic markers, such as serum undercarboxylated osteocalcin (ucOC), tartrate-resistant acid phosphatase 5b (TRACP-5b), and procollagen type 1 N-terminal propeptide (P1NP), represents another limitation. Including these markers in future studies could provide valuable insights into the underlying bone metabolic changes associated with ADT. Finally, our study evaluated L2, L3, and L4 individually rather than as a whole to observe longitudinal changes in BMD related to bone metastases. The Japanese guideline recommends excluding data when only a single vertebral body can be evaluated^14^, as its purpose is to determine the need for osteoporosis treatment. However, our approach, while not suitable for therapeutic decision-making, was tailored to minimize the influence of pathological changes and focus on research objectives.

## Conclusion

The 1-year changes in BMD at non-bone metastatic sites in prostate cancer patients with bone metastases and at the same sites in patients without bone metastases were similar. Our findings suggest that monitoring BMD at non-metastatic sites may be suitable for measuring the effect of systemic therapy on bone density for patients with bone metastasis.

## Supplementary Information

Below is the link to the electronic supplementary material.

Supplementary file1 (PPTX 40 KB)

## Abbreviations

ADT: Androgen deprivation therapy
ARSI: Androgen-receptor signaling inhibitors
BMD: Bone mineral density
DXA: Dual-energy X-ray absorptiometry

## References

[1] Goode EA, Wang N, Munkley J (2023) Prostate cancer bone metastases biology and clinical management (review). Oncol Lett 25:163 36960185 10.3892/ol.2023.13749 PMC10028493

[2] Culig Z, Steiner H, Bartsch G et al (2005) Mechanisms of endocrine therapy-responsive and -unresponsive prostate tumours. Endocr Relat Cancer 12:229–244 15947099 10.1677/erc.1.00775a

[3] Baldessari C, Pipitone S, Sabbatini R et al (2023) Bone metastases and health in prostate cancer: from pathophysiology to clinical implications. Cancers (Basel) 15:1518 36900309 10.3390/cancers15051518 PMC10000416

[4] Nguyen PL, Alibhai SMH, Smith MR et al (2015) Adverse effects of androgen deprivation therapy and strategies to mitigate them. Eur Urol 67:825–836 25097095 10.1016/j.eururo.2014.07.010

[5] Greenspan SL, Coates P, Sereika SM et al (2005) Bone loss after initiation of androgen deprivation therapy in patients with prostate cancer. J Clin Endocrinol Metab 90:6410–6417 16189261 10.1210/jc.2005-0183

[6] Wang A, Obertová Z, Brown C et al (2015) Risk of fracture in men with prostate cancer on androgen deprivation therapy: a population-based cohort study in New Zealand. BMC Cancer 15:837 26525985 10.1186/s12885-015-1843-3 PMC4631090

[7] Mussolino ME, Gillum RF (2008) Low bone mineral density and mortality in men and women: the Third National Health and Nutrition Examination Survey linked mortality file. Ann Epidemiol 18:847–850 18809342 10.1016/j.annepidem.2008.07.003 PMC2640226

[8] Fukumoto S, Soen S, Matsumoto T et al (2020) Subcommittee for CTIBL in the JSBMR. Management manual for cancer treatment-induced bone loss (CTIBL): position statement of the JSBMR. J Bone Miner Metab 38:141–144 32020289 10.1007/s00774-020-01087-0

[9] Coleman R, Body JJ, Aapro M et al (2014) Bone health in cancer patients: ESMO Clinical Practice Guidelines. Ann Oncol 25(Suppl 3):124–137 10.1093/annonc/mdu103 24782453

[10] Berruti A, Dogliotti L, Tarabuzzi R et al (2002) Changes in bone mineral density, lean body mass and fat content as measured by dual energy x-ray absorptiometry in patients with prostate cancer without apparent bone metastases given androgen deprivation therapy. J Urol 167:2361–2367 11992038

[11] Maillefert JF, Sibilia J, Michel F et al (1999) Bone mineral density in men treated with synthetic gonadotropin-releasing hormone agonists for prostatic carcinoma. J Urol 161:1219–1222 10081873

[12] Hara T, Nishimoto H, Miyake H et al (2024) Temporal declines in bone mineral density and trabecular bone score during androgen deprivation therapy. J Bone Miner Metab 42:668 39266779 10.1007/s00774-024-01537-z

[13] Jacob A, Raj R, Allison DB et al (2021) Androgen receptor signaling in prostate cancer and therapeutic strategies. Cancers (Basel) 13:5417 34771580 10.3390/cancers13215417 PMC8582395

[14] Soen S, Fukunaga M, Sugimoto T et al (2013) Diagnostic criteria for primary osteoporosis: year 2012 revision. J Bone Miner Metab 31:247–257 23553500 10.1007/s00774-013-0447-8

[15] Kanda Y (2013) Investigation of the freely-available easy-to-use software “EZR” (Easy R) for medical statistics. Bone Marrow Transplant 48:452–458 23208313 10.1038/bmt.2012.244 PMC3590441

[16] Bagi C (2003) Skeletal implications of prostate cancer. J Musculoskelet Neuronal Interact 3:112–117 15758351

[17] Hussain A, Tripathi A, Guise T et al (2021) Bone health effects of androgen-deprivation therapy and androgen receptor inhibitors in patients with nonmetastatic castration-resistant prostate cancer. Prostate Cancer Prostatic Dis 24:290–300 33028943 10.1038/s41391-020-00296-y PMC8134041

[18] Jones C, Gray S, Sachdeva A et al (2024) Risk of fractures and falls in men with advanced or metastatic prostate cancer receiving androgen deprivation therapy and treated with novel androgen receptor signaling inhibitors: a systematic review and meta-analysis of randomized controlled trials. Eur Urol Oncol. 7:993 38383277 10.1016/j.euo.2024.01.016

[19] David K, Devos G, Deboel L et al (2023) Changes in bone and mineral homeostasis after short-term androgen deprivation therapy with or without androgen receptor signalling inhibitor—substudy of a single-centre, double blind, randomised, placebo-controlled phase 2 trial. EBioMedicine 97:104817 37804569 10.1016/j.ebiom.2023.104817 PMC10570709

[20] Guilhem R, Marie K, Karim F et al (2022) Bone mineral density in men with de novo metastatic castration-sensitive prostate cancer treated with or without abiraterone plus prednisone in the PEACE-1 phase 3 trial. J Clin Oncol 40(6_Suppl):19–19

[21] Perk H, Yildiz M, Ozorak A et al (2008) Correlation between BMD and bone scintigraphy in patients with prostate cancer. Urol Oncol 26:250–253 18452814 10.1016/j.urolonc.2007.05.027

