# Supplementary material for: Longitudinal assessment of bone mineral density in prostate cancer patients: comparing metastatic and non-metastatic regions
Source: Int J Clin Oncol. 2025 Feb 7;30(4):797–804. doi: 10.1007/s10147-025-02711-7 (PMC11946959; doi:10.1007/s10147-025-02711-7)
Supplement: Supplementary file 1 — Supplementary file1 (PPTX 40 KB) [file 10147_2025_2711_MOESM1_ESM.pptx]

## Slide 1
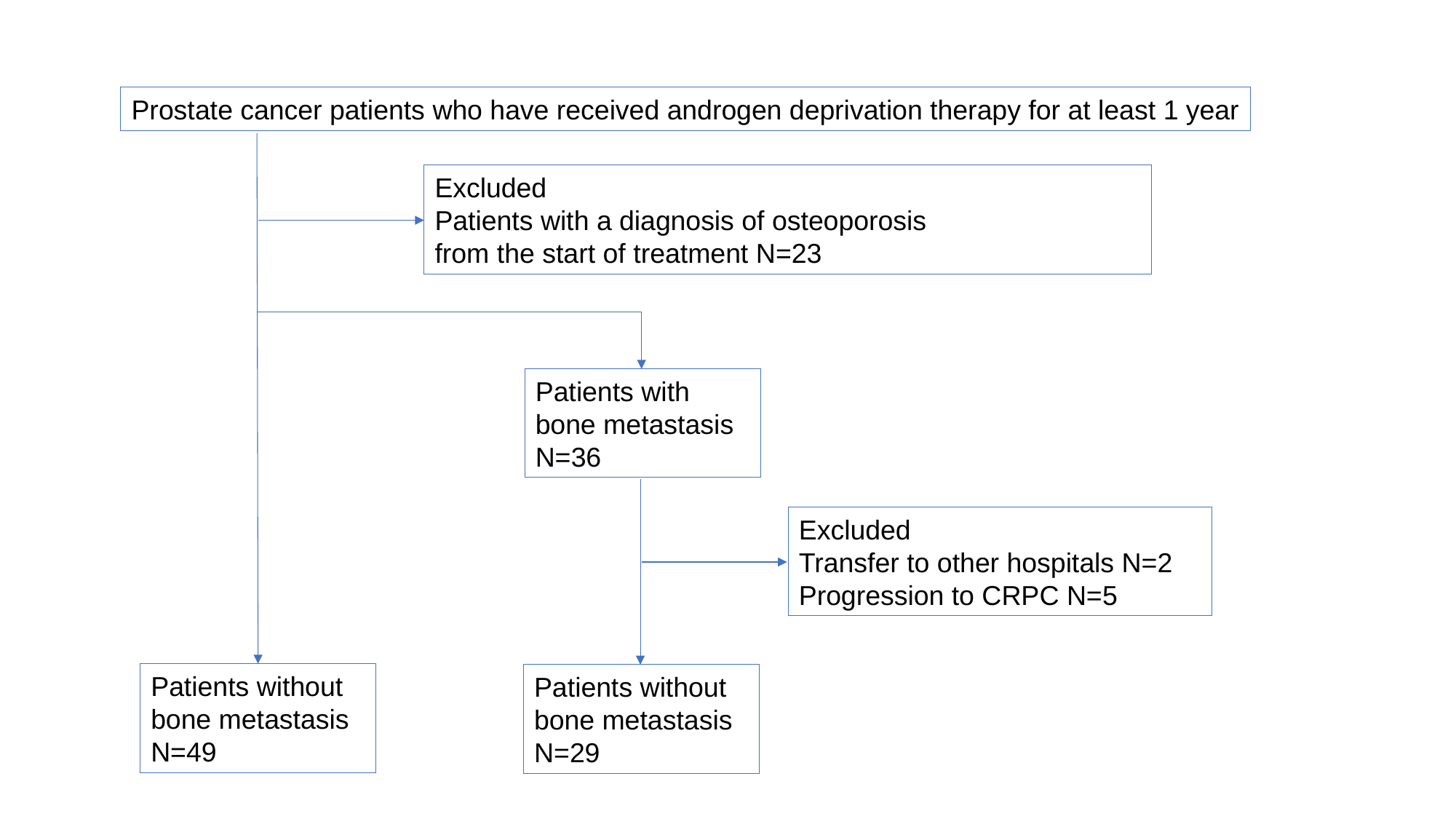

Prostate cancer patients who have received androgen deprivation therapy for at least 1 year
Excluded
Patients with a diagnosis of osteoporosis
from the start of treatment N=23
Patients with
bone metastasis
N=36
Excluded
Transfer to other hospitals N=2
Progression to CRPC N=5
Patients without
bone metastasis
N=49
Patients without
bone metastasis
N=29
